# Solving Groundwater Flow Problems with Time Series Analysis: You May Not Even Need Another Model

**DOI:** 10.1111/gwat.12927

**Published:** 2019-07-25

**Authors:** Mark Bakker, Frans Schaars

**Affiliations:** ^1^ Artesia Water, Korte Weistraat 12, 2871 BP Schoonhoven The Netherlands

## Abstract

Time series analysis is a data‐driven approach to analyze time series of heads measured in an observation well. Time series models are commonly much simpler and give much better fits than regular groundwater models. Time series analysis with response functions gives insight into why heads vary, while such insight is difficult to gain with black box models out of the artificial intelligence world. An important application is to quantify the contributions to the head variation of different stresses on the aquifer, such as rainfall and evaporation, pumping, and surface water levels. Time series analysis may be applied to answer many groundwater questions without the need for a regular groundwater model, such as what is the drawdown caused by a pumping station? Or, how long will it take before groundwater levels recover after a period of drought? Even when a regular groundwater model is needed to solve a groundwater problem, time series analysis can be of great value. It can be used to clean up the data, identify the major stresses on the aquifer, determine the most important processes that affect flow in the aquifer, and give an indication of the fit that can be expected. In addition, it can be used to determine calibration targets for steady‐state models, and it can provide several alternative calibration methods for transient models. In summary, the overarching message of this paper is that it would be wise to do time series analysis for any application that uses measured groundwater heads.

## Introduction

The objective of this issue paper is to discuss the application of time series analysis of measured heads. Time series analysis is a data‐driven approach to find a relationship between input and output signals. In hydrogeology, a relationship is sought between measured heads in an observation well and the measured stresses on the aquifer, such as rainfall and pumping. Time series analysis can be applied to answer many groundwater questions and it is much easier and quicker to apply than a regular, spatial groundwater model. But even for questions that require a spatial groundwater model, time series analysis is an indispensable technique to analyze heads before the development and calibration of a groundwater model. In this paper, it is first reviewed why heads are measured and what the advantages are of data‐driven modeling. Next, the method of response functions is discussed in more detail. It is discussed what problems can and cannot be solved with time series analysis, what the challenges are in modeling time series of heads, and how time series analysis can be applied in combination with regular groundwater models. Finally, a short example of time series analysis is presented to demonstrate how the effect of a pumping well can be identified when the head variations are dominated by tides and rainfall.

### Why Measure Heads?

Heads are measured in many observation wells around the world. The coverage is very uneven: in some areas there is a high density of wells, while in other areas there are hardly any. In addition, measurement frequencies vary (sometimes for the same well), observation periods may be long or short, and there may be gaps in the recording periods. In most observation wells, heads go up and down continuously due to a number of inputs, variably referred to as stresses, drivers, or forces. The major stresses are rainfall, evaporation, surface water levels, pumping, and barometric variations.

Observation wells are installed to measure heads for a variety of reasons. Some are installed to quantify the seasonal behavior of the head, or to monitor long‐term changes or trends in an aquifer. Others are installed to determine the effect of one specific stress on an aquifer, for example, the decline and recovery caused by a period of drought followed by a period of rainfall, or the drawdown caused by a well field. The measured head variation is the combined effect of all the stresses on the aquifer. The effect of a single stress can only be determined by quantifying the contribution of all significant stresses on the aquifer. For many applications, such quantification needs to be accompanied by an estimate of the uncertainty.

It is difficult to assess the actual information content of a time series of head observations. Measured head series represent a smoothed‐out response to all the stresses on the aquifer and may not contain enough information to answer the question under consideration. The information content of a time series of heads is a function of the behavior of the system and of the number of significant stresses that act on the aquifer, the variation of these stresses, and the correlation between these stresses. For example, increased evaporation in summer often coincides with an increase in pumping, which can make it difficult to differentiate between the effect of evaporation and the effect of pumping.

### Data‐Driven Modeling

The analysis of a time series of measurements is a common task in many fields. In this paper, the focus is on the subset of methods that are referred to as system identification, which is the study of models that translate input signals into output signals (e.g., Wang and Garnier 2011). In hydrogeology, such identification and quantification are traditionally carried out with groundwater models. Groundwater models are analytical or numerical solutions to (systems of) differential equations that describe the flow of groundwater. Application of groundwater models requires detailed knowledge (or rough approximations) of both the subsurface and the physical boundary conditions that cause the flow, and take a significant amount of time to develop. These models are also referred to as white box models.

In contrast to white box models, there are gray box models and black box models. For either shade of gray, there is no need for experimental (Darcy's law) or physical (continuity) laws, let alone complicated differential equations, detailed geological models, or GIS coverages of spatial data. Instead, the entire analysis is data driven. Gray box and black box models try to determine a (mathematical) relation between input series and output series (for hydrogeology: a relation between time series of stresses on the aquifer and time series of measured heads in an observation well) without going into (too much) detail about what is actually happening in the aquifer.

Formal definitions of gray box vs. black box models differ. Here, black box models are defined as models that apply some algorithm to translate the input signal into the output signal, without any worries about the underlying physics. Most methods for system identification are black box models from the artificial intelligence world, but they rarely provide physical insight into why the heads vary the way they do (e.g., Siegel and Hinchey 2019). Gray box models, also referred to as semi‐physical models, are models that apply algorithms that have some physical basis. The operative word here is “some,” which can mean as little as that the heads go up when it rains and heads eventually go down when it stops raining. Gray box models have a limited number of parameters that can be tweaked to fit the modeled signal to the measured signal. In the groundwater literature, application of gray box and black box models for system identification is often referred to as time series analysis, while this term is rarely used for white box models even when applied to simulate transient groundwater flow. Nevertheless, the term “time series analysis” will be used in this paper.

Popular black box models include Box‐Jenkins type models (e.g. Changnon et al. 1988; Gehrels et al. 1994; Van Geer and Zuur 1997), which originate from economics (e.g., Box and Jenkins 1970), artificial neural networks (e.g., Daliakopoulos et al. 2005), and other artificial intelligence approaches (e.g., Sahoo et al. 2017; Wunsch et al. 2018). An increasingly popular gray box model is a method that makes use of predefined, physically realistic response functions (e.g. Von Asmuth et al. 2002). Some may even argue that a simple groundwater model can be considered a (light) gray box model (e.g., Obergfell et al. 2016).

Time series analysis is often seen as a statistical technique where output is accompanied by an estimate of the uncertainty. The residuals (the difference between the modeled and measured values) of a transient groundwater model are commonly serially correlated, where modeled heads are above the measured heads for a period, followed by a period where they are below the measured heads. Estimation of uncertainty for models with serially correlated residuals is challenging. One option that is frequently used in time series analysis is to model the serially correlated residuals with an appropriate noise model. Performance of noise models varies, however, as they do not always result in white noise.

Time series analysis requires the availability of measured head series and measured (or estimated) stresses. The longer a time series, the better. Time series of the stresses preferably start before the time series of heads, as the head measurements in the first part of a time series are a function of what happened before the first head measurement. The process of time series analysis consists of three steps: select a model structure, apply an estimation method to estimate the parameters of the model, and evaluate the model results to determine whether the model is adequate for its intended application.

In the remainder of this paper, the pros and cons are discussed of time series analysis of heads with gray box models using predefined response functions. Many groundwater questions can be answered with time series analysis, without ever having to build a regular groundwater model. Other questions can only be answered with a regular groundwater model, but even then, time series analysis can be of great value. In the following, it is explained what time series analysis with response functions is, how the method can be extended with other linear or nonlinear concepts, what kind of problems can and cannot be solved, and how time series analysis can be used to improve regular groundwater models.

### Time Series Analysis with Response Functions

**Figure 1:**
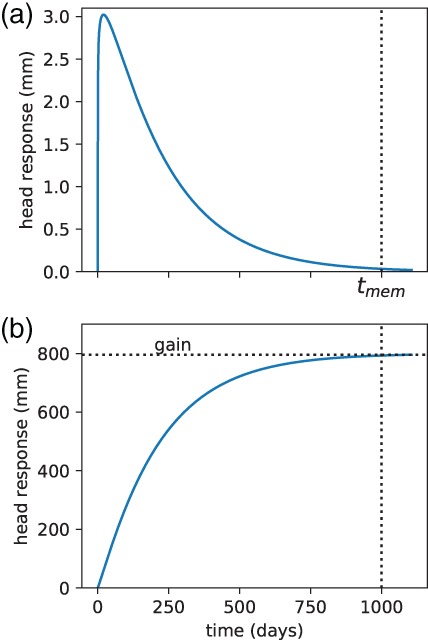
Example response functions. (a) Block response to 1 mm of rainfall in day 1. (b) Step response to 1 mm/days of rainfall starting at *t* = 0.

The basic idea of a time series model based on response functions is that the head in an observation well is the sum of the individual effects on that head from the different stresses that act on the aquifer. Successful application of this method results in estimation of the response function of each stress, including an estimate of the uncertainty (if possible), and the contribution of each stress to the total head variation. The response function represents the relationship between the variation in the stress and the variation in the head. An example of the head response due to 1 day of rainfall, called the block response, is shown in Figure 1a. As expected, the head goes up after the rain starts and eventually comes back down when it stops raining. The time between the start of the rainfall and the time when the head is approximately back to where it started is called the memory, *t*_mem_, of the system (Figure 1). The head response due to continuous rainfall starting at time *t* = 0 is shown in Figure 1b; this is called the step response. When it rains at a constant rate for a long time, the head eventually reaches a plateau. This plateau is called the gain of the response function (Figure 1b). The head at a point in time is a function of all the rainfall in the past *t*_mem_ days and can be obtained through convolution of the time series of rainfall with the response function (superposition in time, e.g., Von Asmuth et al. 2002).

In time series modeling with response functions, sets of physically realistic shapes are selected to estimate the head response for a specific stress. Each response function has a few shape parameters that can be tweaked to optimize the fit between the measured and modeled heads. Different stresses can have different types of response functions. For example, the response to recharge can often be described with a scaled Gamma distribution with three parameters (e.g. Besbes and de Marsily 1984; Von Asmuth et al. 2002). Analytic solutions to simple groundwater flow problems are also good candidate response functions. For example, the Hantush function can be used to simulate the head response of pumping wells (e.g., Von Asmuth et al. 2002). The use of analytic solutions of simple flow problems as response functions has the additional benefit that model parameters can be converted into estimates of aquifer parameters. Care must be taken, of course, that such a conversion only makes physical sense if the analytic solution is a reasonable representation of the actual aquifer system.

Time series analysis with response functions often gives a very good fit to the data, commonly much better than can be obtained with a regular groundwater model. Time series models with response functions are relatively simple, as they include only (a few) handful of parameters. The estimated response functions give modelers physical insight into why heads vary the way they do.

Time series analysis using response functions works well for systems that are sufficiently linear. Nonlinear processes may be added when they play an important role. One nonlinear process that may be important is the nonlinear relationship between measured rainfall and reference evaporation on the one hand and groundwater recharge on the other hand. This may be the case in aquifers with thick unsaturated zones, in areas with significant runoff, or in arid regions (e.g., Berendrecht et al. 2006; Peterson and Western 2014). Another common nonlinear process is when the head response is a function of the head itself. An example is a system where surface water features (ditches, drains, streams) carry water only part of the year, so that the response function changes over time depending on the head in the aquifer (e.g., Knotters and de Gooijer 1999). These are just two examples of nonlinear processes for which potential solutions have been developed in a time series framework, but there are many other nonlinear processes for which solutions are not yet available.

### What Problems Can Be Solved with Time Series Analysis?

A common application of time series analysis in hydrogeology is to detect or quantify a step or a trend in the observed heads. Specialized techniques exist for step and trend detection, including techniques that are designed to find trends when they are obscured by seasonal fluctuations (e.g., Helsel and Hirsch 2002). In the framework of time series analysis, comprehensive step and trend analyses can be conducted that take into account all stresses on the system. A separate step or trend may be added to the model if the stresses in the time series model are not able to simulate the observed step or trend. In such a case, further investigation is needed to identify the cause of the step or trend. Steps and trends may be caused by a number of interventions varying from land‐use change to an unknown increase in pumping. Steps may also simply be measurement artifacts, for example, when a pressure transducer is unintentionally reinstalled at a different depth or a land survey has resulted in an updated measurement of the elevation of the top of an observation well. Another common application of time series analysis is to quantify the long‐term temporal or seasonal variability of the head from shorter time series. Especially in areas with shallow groundwater tables, the temporal variability can be of great economic or ecological importance (e.g., Von Asmuth and Knotters 2004).

Head measurements reflect the combined effect of all stresses on the aquifer. Time series analysis really shines when it is applied to unravel the measured head series to quantify the effect of individual stresses. Many practical questions can be answered with this approach. For example: What is the drawdown caused by a pumping station? How long will it take before the heads recover after a long period of drought? How high will heads rise after a period of high rainfall? What is the effect of changes in rainfall patterns, for example, due to changes in the climate? As with any groundwater model, forecasting of heads outside the measured range of head variations is uncertain, unless it is known that the behavior of the system will not change. Examples of changes in the system behavior are surface water ponding as a result of increased rainfall, or when an increase in pumping near a river causes a disconnect between the groundwater table and the river.

Many groundwater questions cannot be answered with time series analysis, even if data is available, but require the development of a spatial groundwater model. Extensive discussions of the groundwater problems that can be solved with spatial groundwater models can be found in standard texts on groundwater modeling. For example, Anderson et al. (2015) list a representative sampling of eight groundwater questions that can be answered with a groundwater model in the introduction of their book on applied groundwater modeling. Several of these questions clearly need a spatial model such as questions about the capture area of a well field or the travel time of a contaminant, while others can be solved with time series analysis such as questions about the effect of pumping, water diversions or climate change on groundwater levels. Questions about changes to the discharge into wetlands or surface water bodies can potentially be answered with time series analysis, but such data are rarely available while spatial groundwater models provide estimates of both head and flow.

### Challenges of Modeling Time Series of Heads

Two challenges in transient groundwater modeling are how to assess the goodness of fit of the model and how to improve the model fit. These two challenges hold just as well for white box models (regular groundwater models) as for gray or black box models. Many metrics exist to express the goodness of fit of a model or to assess whether a model meets conditions to draw statistical conclusions. Two of the most common goodness‐of‐fit metrics are the root mean squared error (RMSE) and the percentage of variance explained (also known as the coefficient of determination or the Nash‐Sutcliffe coefficient).

If a time series analysis results in a poor fit, which will happen regularly, the modeler is faced with the challenge to determine why a poor fit is obtained. After making sure that no elementary mistakes are made in the analysis (input error, nonconverging solution, etc.), the main suspects are (note that these challenges are common to both regular groundwater models and time series models):

1. Inaccurate head measurements, for example, poorly installed or leaky observation wells, or problems with automatic pressure transducers (e.g., Post and von Asmuth 2013).
2. Missing or incomplete stresses, for example, undocumented and/or illegal pumping, unknown surface water level variations, a regional decline in heads caused by an overall increase in pumping, or stresses that are not measured before the change that is to be evaluated.
3. Inaccurate stresses, for example, poor well discharge data, rainfall estimates from a weather station that is too far away.
4. Overparametrization, for example, not enough data and/or too complex a model to estimate the parameters.
5. Convergence to a local optimum, for example, the parameter estimation algorithm finds a local optimum but misses the global optimum.
6. Missing processes, for example, recharge is simulated as a linear function of measured rainfall and reference evaporation while the relationship is nonlinear, or drainage systems prevent the head from rising above the level of the drains.
7. A change in the system behavior, for example, land‐use change has changed the relationship between rainfall, reference evaporation, and recharge. Or a stream is dredged or deepened, as such removing or reducing the leaky stream bed, resulting in a different response function (Obergfell et al. 2019).

As stated, all these challenges hold for both time series analysis and regular groundwater modeling; only the latter two may be easier to resolve with regular groundwater models. The advantage of time series analysis is that the modeler is constantly reminded of these challenges, as the vast majority of the modeling work consists of comparing measured and modeled heads and trying to improve the model fit. In regular groundwater models, there may be many other reasons why a poor fit is obtained, including conceptualization of the subsurface, spatial heterogeneity, and applied boundary conditions (see e.g., Anderson et al. 2015), which means that equifinality is lurking.

### Application of Time Series Analysis in Combination with Regular Groundwater Models

Head measurements are at the core of any groundwater model calibration. Measured heads need to be analyzed thoroughly prior to the start of calibration. The first step in any data analysis is to visualize the data. Visual inspection gives a first impression whether the measured heads make any sense. Is the seasonal variation realistic? Are there gaps in the data? Are there obvious outliers? Next, time series analysis can be applied to detect, quantify, and resolve many of these issues. If time series analysis results in a good fit, it can be used to detect errors and identify outliers (e.g., Peterson et al. 2018), to fill gaps (e.g., Peterson and Western 2018), or to extend series towards the past or future.

A time series model gives a good indication of the suitability of a measured head series for the calibration of a groundwater model. If a good fit is obtained, it is known which stresses must be included in the model, which processes are needed in the model, and what kind of fit is possible prior to the start of calibration. As stated, time series models commonly give a much better fit than regular groundwater models. If a bad fit is obtained with a time series model, it is likely that a groundwater model based on the same stresses will not do much (if any) better, and the modeler should be cautious in using that head series for calibration, as the model is not a good representation of what happens in the aquifer. Only if the groundwater model includes significant (nonlinear) processes that are not included in the time series model, there may be some hope for a successful calibration.

Upon successful completion of time series analysis at several observation wells, a spatial map of characteristics of the estimated response functions may provide additional information to the modeler. For example, a map of the gain of the response to recharge (the steady increase of the head as a result of a uniform stress) should result in a logical pattern: for example, a higher gain between streams and a lower gain near streams. In the absence of such a logical pattern, it will be difficult to calibrate a regular groundwater model, which also produces logical patterns. Similarly, if the gain caused by rainfall in an observation well next to a stream is significant, that is an indication that the groundwater is not in full contact with the stream, and a leaky stream bed should be included in the groundwater model.

For steady‐state groundwater models, time series analysis can be applied to determine representative steady‐state values from a time series of head observations, including corresponding steady‐state stress values. This is even more valuable when the observation periods differ between observation wells (they do not even need to overlap), or when the observation frequency changes. Time series analysis can provide an uncertainty estimate for the calculated steady response if conditions for statistical inference are met, which allows the modeler to assign weights in the calibration process, based on the estimated uncertainty. Time series analysis may also be used to estimate the steady response for one stress only, leaving out the other stresses. These estimates may be used as calibration targets for a model of that one specific stress only.

Time series analysis provides alternative opportunities to calibrate transient groundwater models when the response functions of the different stresses are estimated. First, a transient model may be calibrated by using the estimated response functions at different observation wells as calibration targets. This way a few relatively short transient model runs are sufficient to calibrate a transient model, rather than a transient calibration over the entire observation period. Second, characteristics of the response functions can be used to calibrate transient groundwater models by calibrating a few steady models (Bakker et al. 2008; Obergfell et al. 2013), including the gain and the mean of the response function. Third, and similar to steady models, a transient model can be calibrated on the transient response of one single stress (e.g., pumping) so that it is not obfuscated by the other stresses like the weather, even if they have a larger contribution to the head.

### Example: Pumping Test in a Tidal Area

**Figure 2:**
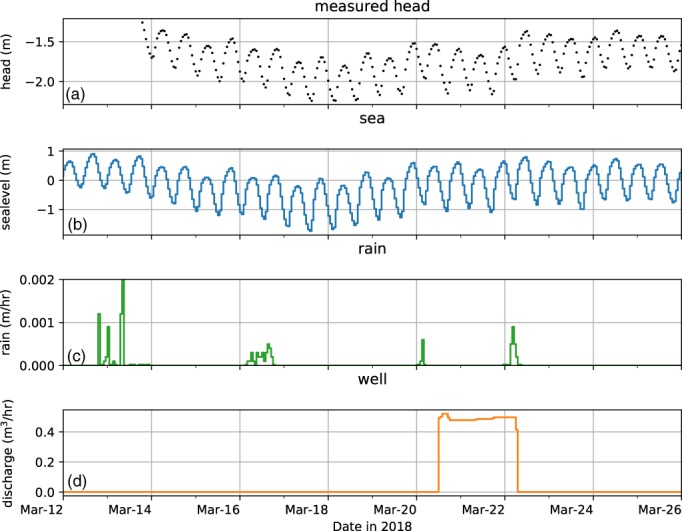
Measured time series of the example: (a) heads, (b) sea level, (c) rainfall, and (d) discharge of the pumping well.

As an example application of time series analysis, consider a pumping test where the pumping response needs to be unraveled from the measured head variation. The pumping test is conducted in the coastal area of the Netherlands, where high artificial sand dunes are constructed to protect the coastal zone from the effects of sea‐level rise. Artificial sand dunes are more natural and flexible compared to conventional dikes. A mixture of sand and saltwater is dumped on the beach by a hopper dredger through a floating pipeline or by using the rainbowing technique. Either way, a vast amount of seawater is deposited on the beach, which may result in saltwater intrusion and a short‐term rise of the groundwater table inland during construction. A pumping test is conducted to estimate aquifer parameters to be used in a regular groundwater model. The head response is measured in 20 observation wells, of which one is analyzed here. Three time series of stresses are involved: the tidal fluctuation of the sea, a few rainfall events, and the measured discharge of the pumping well (Figure 2). The head is measured hourly for a period of 12 days while the pump is on for only 2 days. All the stresses are available prior to the period of head measurements (Figure 2b to 2d).

The head variation is dominated by the tide and the effect of the pumping test is difficult to see in the head measurements (Figure 2a). Time series analysis with response functions is used to extract the pumping response from the measured heads, using the open source code Pastas (Collenteur et al., 2019). Three models are developed with an increasing number of stresses to evaluate the improvement of the model fit due to each additional stress. For this simple setup, the response function of each stress is an exponential function with just two parameters. The RMSE, explained variance, and number of parameters of each model are listed in Table 1.

**Table 1:** Results of Time Series Models of the Example for Three Models with an Increasing Number of Stresses

Stresses	RMSE (m)	Explained Variance (%)	Model Parameters
Sea	0.08	87.5	3
Sea + Rain	0.07	91.0	5
Sea + Rain + Well	0.05	95.1	7

**Figure 3:**
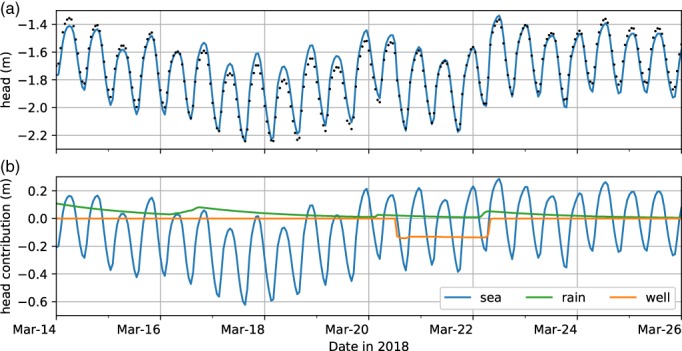
Results of time series analysis of the example (a) measured and simulated heads using all three stresses and (b) separate head contributions of the three stresses.

The first model only includes the sealevel as a stress and results in an explained variance of 87.5%, but the simulated heads are lower than the measured heads at the beginning of the measurement period and higher than the measured heads during the time of pumping (not shown). Next, rainfall is added as a stress and the explained variance increases to 91%. Addition of rainfall to the model improves the model fit in the first days of the measurement period, but the simulated heads are still too high during pumping. Finally, pumping is added and the explained variance increases to 95.1%. The model fit is now good over the entire measurement period. The model fit and the contributions of the three stresses to the head variation are shown in Figure 3. Addition of the three contributions of Figure 3b plus a constant gives the simulated head in Figure 3a. The estimated head response due to the pumping test is estimated between 12 and 14 cm in this observation well.

Time series analysis was conducted for all 20 observation wells to isolate the contribution of the pumping test. The gains at the observation wells show a logical pattern (both horizontally and vertically) and were used in a steady‐state analytical model to estimate the aquifer parameters (not shown). The aquifer parameters were implemented in a numerical transport model to predict the impact of the construction of the dune. This example demonstrates how time series analysis may be used to extract the pumping response from a measured head series even though it is barely visible in the measured heads due to the effects of the other stresses on the model.

## Conclusion and Discussion

Heads are measured in many observation wells and for a variety of reasons, periods, and frequencies. Time series analysis is a relatively simple data‐driven approach to analyze measured heads, and, when response functions are used, gives insights into why the heads vary the way they do. Meaningful analysis of measured heads requires knowledge of the different stresses that cause the head variation. Time series analysis may be used to answer many groundwater questions without ever having to build a regular groundwater model and many series of head observations can be analyzed in batch.

Time series analysis is much quicker than the development of a regular groundwater model. Each model is relatively simple with a small number of parameters and very few additional approximations. This in contrast to regular groundwater models, which require extensive approximations and parameterizations of the subsurface and boundary conditions, all with their own uncertainty. A much better fit is commonly obtained with time series analysis than with a regular groundwater model, despite all the additional information that is entered in the latter.

Not all time series models give a good fit. A poor fit may indicate that some stresses are missing or that some important processes are not included. These stresses and or processes need to be identified before a groundwater problem can be solved. It may even be questioned whether it makes sense to keep on measuring heads if the stresses that are causing the head variation are not measured simultaneously.

Many groundwater questions can only be answered with a regular groundwater model. In such a case, time series analysis can still play an important role. Time series analysis can clean up the data, can give an indication of how good a fit can be expected with a regular groundwater model, and what stresses and processes need to be included. In addition, it can provide representative calibration targets for steady groundwater models or provide efficient alternatives for calibration of transient groundwater models.

There are several tools for time series analysis of heads, including the commercial Menyanthes (Von Asmuth et al. 2012), the open source matlab code Hydrosight (Peterson and Western 2014), and the open source Python code Pastas (Collenteur et al., 2019). Methods for time series analysis are actively being developed to enhance its capabilities in the future.
